# Cepred: Predicting the Co-Expression Patterns of the Human Intronic microRNAs with Their Host Genes

**DOI:** 10.1371/journal.pone.0004421

**Published:** 2009-02-10

**Authors:** Dong Wang, Ming Lu, Jing Miao, Tingting Li, Edwin Wang, Qinghua Cui

**Affiliations:** 1 Department of Medical Informatics, Peking University Health Science Center, Beijing, China; 2 MOE Key Laboratory of Molecular Cardiology, Peking University, Beijing, China; 3 Department of Pediatrics, Peking University First Hospital, Beijing, China; 4 MOE key laboratory of Bioinformatics, Tsinghua University, Beijing, China; 5 Computational Chemistry and Biology Group, Biotechnology Research Institute, National Research Council Canada, Montreal, Québec, Canada; University of Glasgow, United Kingdom

## Abstract

Identifying the tissues in which a microRNA is expressed could enhance the understanding of the functions, the biological processes, and the diseases associated with that microRNA. However, the mechanisms of microRNA biogenesis and expression remain largely unclear and the identification of the tissues in which a microRNA is expressed is limited. Here, we present a machine learning based approach to predict whether an intronic microRNA show high co-expression with its host gene, by doing so, we could infer the tissues in which a microRNA is high expressed through the expression profile of its host gene. Our approach is able to achieve an accuracy of 79% in the leave-one-out cross validation and 95% on an independent testing dataset. We further estimated our method through comparing the predicted tissue specific microRNAs and the tissue specific microRNAs identified by biological experiments. This study presented a valuable tool to predict the co-expression patterns between human intronic microRNAs and their host genes, which would also help to understand the microRNA expression and regulation mechanisms. Finally, this framework can be easily extended to other species.

## Introduction

MicroRNA (miRNA) is a class of small non-coding RNAs (∼22 nt) identified in recent years. They usually function as the negative regulators for certain genes at the post-transcriptional level by binding to the 3′UTRs of the target mRNAs through base pairing, resulting in the target mRNAs cleavage or translation inhibition [1], [2], [3]. It has been newly shown that miRNAs may also function as positive regulators [4], [5]. It is estimated that 1–4% of the genes in human genome are miRNAs, a single miRNA can regulate more than 200 targets [6], and the miRNAs preferentially regulate duplicated genes in mammals [7]. Increasing evidences suggest that the miRNAs play crucial roles in nearly all the important biological processes, such as cell growth, proliferation, differentiation, development, and apoptosis [6]. It has been reported that miRNAs also participate in the cellular signaling networks[8] and the gene regulatory networks [9], as well as the cross-species gene expression variation[10] and pathways [11]. Hereby, the miRNAs might be associated with various diseases [12].

To get a more comprehensive understanding of the miRNAs, it becomes important to identify the tissues in which a miRNA is expressed, which would promote not only the understanding of the miRNA biogenesis and expression mechanisms but also the prediction of their functions. However, it is difficult to identify the tissues in which a miRNA is expressed because great limitations exist in the current large-scale expression profile detecting techniques, such as the microarray [13]. For example, it is difficult to detect the expression levels of those miRNAs in rare cells or expressed at low levels [13]; the amount of miRNAs contained in a microarray chip is relatively limited comparing to the total amount of miRNAs in the human genome. Moreover, microarray experiments are both time and cost consuming. Most importantly, detecting the miRNA expressions of human directly is more difficult than other species since the lack of tissues. Therefore, it becomes critical to develop new approaches to decipher the expression of miRNAs.

miRNAs show a very biased distribution in genomes. More than 36% (196/533) of the human miRNAs are within the introns (referred as the intronic miRNA here) of protein-coding genes (the host genes, miRBase Version 10, October 2007). The enrichment of the intronic miRNAs in human genome triggered researchers to wonder whether the intronic miRNAs and their host genes have special connections in terms of expression and regulation. Indeed, several groups have reported that many intronic miRNAs show significantly correlated expression profiles with their host genes [14], [15], [16], [17]. One possible explanation for this phenomenon is that (1) the intronic miRNAs may be co-transcribed with their host genes by inclusion introns of their pre-mRNAs [17]; (2) the intronic miRNAs and their host genes may share common regulatory elements, such as common promoters [18]. By performing a comparison between the known miRNA expression profiles [19] and the RT-PCR expression profiles of the host genes, Rodriguez et al. found perfect correlations between the expression profiles of the investigated intronic miRNAs and their host genes [17]. These findings seemed to provide us a chance to uncover the tissues in which an intronic miRNA is expressed by associating with the expression profiles of its host gene. However, there are some intronic miRNAs which are low co-expressed with their host genes [14], which makes the problem much more complicated. Therefore, the first step to infer the expression tissues of the intronic miRNAs in this way is to identify which intronic miRNAs show high co-expression with the host genes and which do not.

In this study, we established a machine learning based approach to predict whether an intronic miRNA is high co-expressed with its host gene. We tested our method by the leave-one-out cross validation scheme, a novel independent dataset, and tissue specific miRNAs. Finally, we applied our approach to all the human intronic miRNAs.

## Results

**Figure 1:**
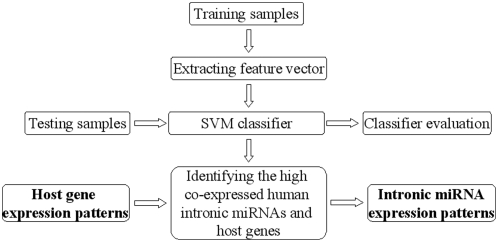
The framework works as follows: A support vector machine (SVM) classifier is trained based on the feature vector extracted from the training samples. And then the performance of this classifier is evaluated by the testing samples. The classifier is further used to identify the human intronic miRNAs that are high co-expressed with their host genes. Finally, the expression profiles of the identified human intronic miRNAs are predicted by inferring that of their host genes.

The co-expression dataset of Baskerville et al. [14] was employed as the training dataset. We first calculated the feature vectors for each sample in the training dataset, which was used to train a classifier. The performance of the classifier was then evaluated. Finally, we applied the classifier to all the human intronic miRNAs. As a result, all human intronic miRNAs showing high co-expression with host genes are identified and then the expression profiles of these miRNAs are inferred by identifying the expression profiles of the host genes. The complete flowchart of our framework is shown in Figure 1.

### Genomic features of the human intronic miRNAs

In this study, the different co-expression patterns of the intronic miRNAs with their host genes triggered us to explore what the reasons are. Zhou et al. reported that the miRNA hosting introns have a 5′-biased relative position distribution compared to all the other introns in human and mouse genomes, suggesting that the cis-signals within the 5′UTRs of the host genes may interfere the transcription and regulation of the intronic miRNAs to certain extent [20]. Moreover, this finding suggests that the relative positions of the intronic miRNAs in their host genes is not completely random and may have functional significance. Therefore, here we present four feature vectors, which reflect the positional characteristics of the intronic miRNAs in their host genes to train the classifier (Table 1). These four features describe the position of miRNA hosting intron, the position of miRNA, the length of the miRNA hosting intron, and the ratio of the length of miRNA to that of the host gene.

**Table 1:** The feature vector of samples used in training the SVM.

Index	Description
1	Distance from the transcription start position of the host gene to the start point of the host intron
2	Distance from the transcription start position of the host gene to the start point of the microRNA
3	Length of the host intron
4	Length of the microRNA/length of the host gene

### Support vector machine training and predicting

A support vector machine (SVM) based classifier was implemented to predict the co-expression levels between the intronic miRNAs and their corresponding host genes. The dataset from Baskerville et . [14] was adopted as the training set. The feature vectors (Table 1) for each sample were computed and are listed in Table 2. As shown in Table 2, columns “1”, “2”, “3”, and “4” list the values of these four features for each miRNA; column “co-expressions” lists the correlation coefficients of the miRNA and host gene expression profiles; column “ER” lists the co-expression patterns of miRNA and host gene identified by biological experiments, in which “+” represents high co-expression and “−” represents low co-expression. A leave-one-out cross validation scheme was employed to evaluate the performance of this SVM classifier. For each round of the leave-one-out experiments, one sample was eliminated from the training set and the remaining samples were used to train the SVM classifier. The prediction was made on the uncovered sample with the well-trained model. Each sample was left out for one time and the prediction results for all the samples are listed in the column “PR” of Table 2. The results of this experiment revealed that our approach achieves an accuracy of 79% and shows good sensitivity and specificity (Table 3), which indicates that the co-expression patterns are predictable using SVM and these four features. The accuracy is calculated as the ratio of the number of the samples that are predicted correctly to the number of the whole samples. The sensitivity of high/low co-expression class is calculated as the ratio of the number of the samples that are predicted as high/low co-expression correctly to the total number of true high/low co-expression samples, respectively. The specificity of high/low co-expression class is calculated as the ratio of the number of the samples that are true high/low co-expression to the number of the samples that are predicted as high/low co-expression, respectively.

**Table 2:** The training dataset and the leave-one-out validation results on Baskerville et al.' data [14].

microRNAs	Host gene	Training dataset*						
		1	2	3	4	co-expressions	ER	PR
hsa-mir-9-1	C1orf61	9933	0.0035	182.716	143.205	0.999	+	+
hsa-mir-139	PDE2A	33502	0.00068	580.925	486.716	0.99	+	+
hsa-mir-1-1	C20orf166	11323	0.00345	55.0571	45.9	0.968	+	+
hsa-mir-95	ABLIM2	24504	0.00041	499.888	228.05	0.96	+	+
hsa-mir-338	AATK	1412	0.00398	138.167	125	0.921	+	+
hsa-mir-126	EGFL7	619	0.00862	91.3452	88.1071	0.888	+	+
hsa-mir-25	MCM7	771	0.0092	9.39759	7.48193	0.838	+	+
hsa-mir-204	TRPM3	43567	0.00128	239.523	3.77982	0.796	+	+
hsa-mir-190	TLN2	12893	0.00043	2100.1	2060.25	0.663	+	+
hsa-mir-153-1	PTPRN	3451	0.0045	50.4157	21.3371	0.626	+	+
hsa-mir-98	HUWE1	2461	0.00097	198.585	187.542	0.503	−	−
hsa-mir-153-2	PTPRN2	5119	0.00008	410.163	377.105	0.499	−	−
hsa-mir-26b	CTDSP1	629	0.01229	38.0526	34.8816	0.453	−	−
hsa-mir-30c-1	NFYC	4874	0.0011	746.761	700.875	0.406	−	−
hsa-let-7f-2	HUWE1	2461	0.00068	297.585	269.878	0.379	−	−
hsa-mir-99a	C21orf34	69554	0.00019	4308.89	4288.26	0.335	−	−
hsa-mir-125b-2	C21orf34	69554	0.00021	4498.4	3898.42	0.297	−	−
hsa-mir-103-2	PANK2	1620	0.00226	359.468	353.701	0.27	−	−
hsa-mir-101-2	RCL1	10574	0.00114	736.718	727.141	0.226	−	−
hsa-let-7c	C21orf34	69554	0.0002	4162.05	4133.27	0.067	−	−
hsa-mir-199a-1	DNM2	7602	0.00061	1419.26	1347.13	0.02	−	−
hsa-mir-32	C9orf5	12165	0.00066	450.652	333.087	−0.22	−	−
hsa-mir-26a-1	CTDSPL	3524	0.00062	1410.88	1390.86	−0.285	−	−
hsa-mir-128a	R3HDM1	13938	0.00042	1652.9	1603.47	0.856	+	−
hsa-mir-103-1	PANK3	2235	0.00321	68.4675	45.8831	0.638	+	−
hsa-mir-15b	SMC4	7415	0.00282	41.5464	40.6804	0.509	−	+
hsa-mir-16-2	SMC4	7415	0.00233	52.3375	49.325	0.504	−	+
hsa-mir-128b	ARPP-21	48416	0.00055	1230.36	1224.2	0.444	−	+
hsa-mir-335	MEST	1633	0.00655	43.172	36.8387	0.442	−	+

* Once again, kicking one sample out as the testing sample, the rest 28 samples are the training dataset.

The four features (columns “1”, “2”, “3”, and “4”) of each miRNA are calculated based on the genomic coordinates of the miRNA, the miRNA hosting intron, and the host gene.

ER represents the experimental results and PR represents the prediction results. The symbol “+” means high co-expression and the symbol “−” means low co-expression.

**Table 3:** Classification performance of the SVM classifier on two testing datasets.

Testing datasets	Type	Size	Accuracy (%)	Sensitivity (%)	Specificity (%)
Baskerville et al.' co-expression data	+	12	79	83	71
	−	17		76	87
The combined data*	+	5	95	100	83
	−	16		94	100

* the combined data is the consistent part of Baskerville et al's miRNA profile data/Su et al.' mRNA profile data and Barad et al.' miRNA profile data/Su et al.' mRNA profile data.

The symbol “+” means high co-expression and the symbol “−” means low co-expression.

In order to confirm the results, we further test our method on an independent dataset. This testing dataset is combined from one set of mRNA microarray data and two sets of miRNA microarray data from three separated labs (see Materials and Methods). There are 5 and 16 intronic miRNAs which showed high or low co-expression patterns with their host genes, respectively (Table 4), in which we evaluated the co-expression patterns of miRNA and host gene by calculating the Pearson's correlation coefficient of miRNA and host gene expression profiles. In this case, we trained the SVM classifier with all samples in Baskerville et al.' data and then applied it on this independent testing dataset. As a result, 20 out of the 21 predicted results turned out to be accordance with the experimental results. More specifically, all the 5 experimental high co-expressed intronic miRNAs, as well as 15 out of the 16 low co-expressed intronic miRNAs were correctly predicted. The missing one was hsa-mir-149, which showed low co-expression in the experimental results but was predicted as high co-expressed by our classifier. Finally, we got a total accuracy of 95% in this validation, which indicates that our approach is robust (Table 3).

**Table 4:** The prediction results on the combined testing data.

miRNAs	Host gene	Pearson's correlation coefficients	Calculated results	Predicted results
hsa-mir-28	LPP	0.2403	−	−
hsa-mir-140	WWP2	−0.279	−	−
hsa-mir-149	GPC1	0.0334	−	+
hsa-mir-23b	C9orf3	0.2401	−	−
Has-mir-194-1	IARS2	−0.4155	−	−
hsa-let-7g	TMEM113	0.6539	+	+
hsa-mir-152	COPZ2	−0.1915	−	−
hsa-mir-93	MCM7	0.7318	+	+
hsa-mir-107	PANK1	−0.254	−	−
hsa-mir-30e	NFYC	0.1416	−	−
hsa-mir-208	MYH7	0.0741	−	−
Has-mir-218-1	SLIT2	0.8282	+	+
Has-mir-106b	MCM7	0.7093	+	+
Has-mir-105-1	GABRA3	0.1756	−	−
hsa-mir-24-1	C9orf3	0.0961	−	−
hsa-mir-215	IARS2	−0.6153	−	−
hsa-mir-214	DNM3	−0.3261	−	−
hsa-mir-186	ZRANB2	0.8033	+	+
hsa-mir-211	TRPM1	0.0692	−	−
Has-mir-199b	DNM1	−0.3855	−	−
hsa-mir-191	C3orf60	0.1413	−	−

The combined data is described as Table 3.

The symbol “+” means high co-expression and the symbol “−” means low co-expression.

### Identifying the tissues in which the human intronic miRNAs are expressed

Using the 29 miRNA-host gene pairs of Baskerville et al., we have constructed a SVM based classifier to predict whether an intronic miRNA is high co-expressed with its host gene. Here we applied this classifier on the rest 178 human intronic miRNAs reported in miRBase database[21] to predict whether they are high co-expressed with their host genes. Finally, 64 and 112 of them were predicted to be high or low co-expressed with their host genes, respectively (Supplementary Text S1). Together with the 12 high co-expressed intronic miRNA-host gene pairs in the training dataset, we obtained 76 high co-expressed intronic miRNA-host gene pairs. The expression profiles of these intronic miRNAs could be estimated by associating with the expression profiles of their host genes. By examining the expression profiles of the host genes across 79 human tissues [22], we obtained the expression profiles of 71 miRNAs in these 79 human tissues (Supplementary Text S2).

### Validation through tissue-specific miRNAs

**Figure 2:**
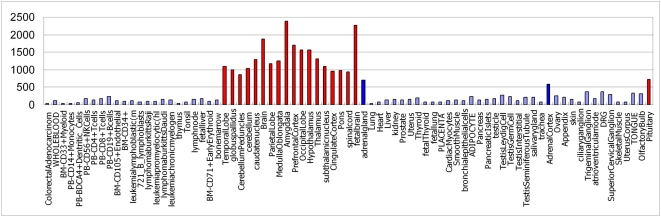
The predicted expression profile of mir-488, which indicates that mir-488 mainly show high expression values in central nervous system. Red and Blue bars represents high expression tissues, in which the red bar represents central nervous system tissues and the blue bar represents adrenal gland and adrenal cortex, respectively.

Among the high tissue-specific miRNAs identified by Landgraf et al. based on small RNA library sequencing [23], 12 of them are intronic miRNAs and show high co-expression with host genes in our prediction. Table 5 lists the specific tissues identified by prediction and biological experiments for these 12 miRNAs, respectively. As a result, 11 of the 12 overlaps were successfully predicted (Table 5), in which mir-302 cluster contains mir-302a, mir-302b, mir-302c, mir-302d, and mir-367. For example, we predicted that hsa-mir-488 is high expressed in the amygdala, temporal lobe, globu spallidus, cerebellum peduncles, cerebellum, caudate nucleus, whole brain, parietal lobe, medulla oblongata, prefrontal cortex, occipital lobe, hypothalamus, thalamus, subthalamic nucleus, cingulated cortex, pons, spinal cord, fetal brain, adrenal gland, adrenal cortex and pituitary, which mainly belong to nervous systems except the adrenal gland and adrenal cortex (Figure 2). As shown in the Figure 2, each bar represents one tissue; the height of the bar represents the expression level of has-mir-488 in that tissue. The tissues with high expression level are highlighted in red and blue color, in which red bars represent tissues of nervous systems.

**Table 5:** Tissue-specific miRNAs that are also found in our predictions.

miRNAs	*Tissues [23]	miRNAs	Predicted tissues
mir-1	Heart	mir-1-1	Skeletal Muscle
mir-488	Nervous system	mir-488	Nervous system
mir-218	Nervous system	mir-218-2	Nervous system
mir-449a	Reproductive System	mir-449a	Ovary
mir-9	Nervous system	mir-9-1	Nervous system
mir-128a	Nervous system	mir-128a	Nervous system
mir-153	Nervous system	mir-153-1	Nervous system
mir-302 cluster	Embryonic tissue and cell lines	mir-302 cluster	lymphoblasts

* The specific tissues listed in this column are identified by biological experiments [23].

The exception is mir-1-1, which was reported to be heart specific by Landgraf et al. and was predicted to show high specificity in skeletal muscle in our study. However, mir-1 was also reported to show high specificity in both heart and skeletal in several other studies, which indicates that all the above 12 predictions in our study are right [24], [25].

**Figure 3:**
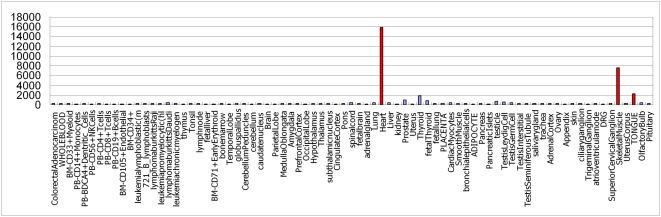
The predicted expression profile of mir-208b, which indicates that mir-208b mainly show high expression values in heart, skeletal muscle, and tongue. Red bars represent high expression tissues. From the left to the right, the three red bars represent heart, skeletal muscle, and tongue, respectively.

Furthermore, we also predicted that hsa-mir-208b shows high specificity in heart, skeletal muscle and tongue (Figure 3). As shown in the Figure 3, each bar represents one tissue; the height of the bar represents the expression level of has-mir-208b in that tissue. The tissues with high expression level are highlighted in red. However, hsa-mir-208b was not identified as tissue specific miRNA by Landgraf et al. [23]. It was reported that hsa-mir-208b show high heart-specificity by Liang et al. [24] and is associated with various cardiovascular diseases [12]. These results indicate that our prediction can provide us valuable assistance in identifying the tissue specific miRNAs.

## Discussion

In summary, we presented a machine learning based approach to predict the co-expression patterns of the human intronic miRNAs and their host genes, which show a high accuracy and validation and could be further extended to other species. We further applied this approach to all the human intronic miRNAs and predicted the tissues in which the miRNAs are expressed for the miRNAs showing high co-expression pattern with their host genes. We also tested the validation of our method by predicting tissue specific miRNAs and performed a comparison with tissue specific miRNAs identified by biological experiments. As a result, our predictions are confirmed to be right. However, limitations exist in our current approach, which could be improved in the future study. We know that the process of miRNA and gene transcription is very complex and could be affected by lots of factors. Therefore, in order to improve the accuracy of the classifier, it will be very important to find better features that are associated with the expression of the intronic miRNAs and their host genes, for example the trans-action elements. For the machine learning methodology, currently we assigned the intronic miRNA-host gene pairs into high co-expression ones and low co-expression ones based on the correlation coefficients between the profiles of the intronic miRNAs and their host genes. This treatment could miss some detailed information, which may be useful in accurately predicting the correlations of the expression profiles between novel intronic miRNAs and their host genes. A regression model may provide more details in this situation. For the sample dataset, the current training dataset is small, which may affect the reliability of the classifier. Therefore, the prediction accuracy could be improved when more training samples become available. Furthermore, the current approach can only deal with the miRNAs that are within the introns of protein-coding genes. Developing computational approaches that can be used to predict the co-expression patterns for other kinds of miRNAs is also important. In a conclusion, our method can provide help in not only the understanding of miRNA expression and regulation but also the function of miRNAs, which can be further used to infer the miRNA-associated diseases.

## Materials and Methods

### The genome coordinate data of human miRNAs

We downloaded the genome coordinate data of the human miRNAs from the miRBase [21] on August 2007 (gff-version 2). This database records 528 items of genome coordinates of human miRNAs. The genome coordinates were used to discriminate whether one miRNA locates within the intron of a protein-coding gene or not.

### The genome coordinates of human protein-coding genes

We obtained the genome coordinates data of known human RefSeq genes through the UCSC (University of California Santa Cruz) Table Browser (http://genome.ucsc.edu/, assembly: Mar. 2006, database: hg18, group: Genes and Gene Prediction Tracks, track: RefSeq Genes, table: refGene) [26]. Each item in this table lists one reference gene and its supplementary information, such as name, chromosome number, strand, transcription start position, transcription end position, coding region start position, coding region end position, number of exons, exon start positions, exon end positions etc. The genome coordinate data of protein coding genes together with that of miRNAs were used to determine whether a miRNA locates within the intron of a protein-coding gene.

### Calculating the genome features of human miRNAs

The four feature vectors for the SVM classifier were calculated from the above genome coordinate data. We first mapped the genome coordinates of human miRNAs and protein-coding genes using Galaxy tool (http://g2.trac.bx.psu.edu/). We then calculated the lengths of all intronic miRNAs, the lengths of miRNA-hosting introns, the lengths of miRNA-hosting genes, the distance from the transcription start position of one host gene to its intronic miRNA, and the distance of the transcription start position of one host gene to the host intron. These parameters will be used as the features of the samples (the miRNA-host pairs).

### The dataset used in training and leave-one-out cross validation

Baskerville et al. performed a human miRNA microarray experiment across 30 normal tissues and investigated the co-expression patterns of 29 pairs of intronic miRNAs and host genes. Some pairs show high co-expression but some others show low co-expression. In the dataset, we set a cutoff of correlation coefficient at 0.6 to discriminate high co-expression and low co-expression. We also performed a leave-one-out cross validation on this dataset.

### The testing dataset

In order to test the generalization power of our approach, we validated the method on an independent testing dataset containing miRNA expression profiles as well as mRNA expression profiles. It has been reported that microarray data from different platforms or laboratories often show great differences. Therefore, to improve the reliability and the persuasion of the results, we adopted two sets of miRNA microarray data. One miRNA microarray dataset was presented by Baskerville et al. [14], the other was obtained from Barad et al. [27]. We downloaded the host gene expression profile from Su's mRNA microarray data which are across 79 normal human tissues [22]. The intronic miRNAs in Baskerville et al.'s dataset and Barad et al.'s dataset were mapped to the host genes in Su's dataset, respectively. We then calculated the Pearson's correlation coefficients between the intronic miRNA expression profiles and their host gene profiles for both miRNA microarray datasets. Only those intronic miRNA-host gene pairs that showed consistent correlation patterns in both two miRNA microarray datasets was reserved for further analysis. As a result, we obtained 21 intronic miRNA-host gene pairs, in which there are 5 high co-expression pairs and 16 low co-expression pairs, respectively (Table 5).

### Support vector machine

Support vector machine (SVM) was introduced by Vapnik [28], and has a comprehensive applications in many classification and regression problems. The goal of SVM is to construct a classifier from well-labeled data (the training data) that can be used to classify the incoming unlabeled data (the testing data). For a binary classification problem, for a given dataset, *x_i_* represents the feature vector, while *y_i_* represents the class labels (*i* = 1,2,…N, where N is the number of samples), where 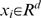, 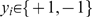. Here, for the intronic miRNA and host gene co-expression prediction problem, the input vector dimension is 5 (d = 5). The class label +1 represents the high co-expression class, while −1 represents the low co-expression class. Once the SVM was trained, it can be used to predict the class label (high or low co-expression) for a new sample (a new intronic miRNA and host gene pair). In this study, the LibSVM package[29] was used to train the SVM intronic miRNA and its host gene co-expression classifier and make predictions in the test dataset. We chose the Radial Basic Function (RBF) as the kernel function and tuned the parameters using the grid search strategy in LibSVM.

### Software availability

We implemented our approach as a web server which is free for the scientific and technical community (http://cmbi.bjmu.edu.cn/cepred/). The “cepred” software in linux and windows are also available for the scientific and technical community when requesting.

## Supporting Information

Text S1 the predicted co-expression patterns of intronic miRNA with their host genes
(0.02 MB XLS)

Text S2 The predicted expression profiles of 71 miRNAs in 79 human tissues.
(0.09 MB XLS)
